# A Practical Manual of Gynecology

**Published:** 1891-09

**Authors:** 


A Practical Manual of Gynecology. By G. R. Southwick, M. D., Assistant Professor of Obstetrics in the Boston University School of Medicine. L. M., Rotunda Hospitals, Dublin. Boston, Otis Clapp & Son, 1891.

That a second edition of this well-known text-book of Gynecology should have appeared is, in itself, a recommendation. We read in the preface: "The author believes that many uterine diseases are largely due to faults either of nutrition or of vascular or nervous supply, and, like other diseases, can be effectually and permanently cured by internal medication." We are glad that the Doctor makes this confession. Still this confession does not quite harmonize with the amount of surgery recommended in the book. It seems our gynecologists are not happy unless they can cut and slash. We are confident that out of one hundred cases of uterine disease fully ninety-five can be cured by internal treatment, without any digital examination, etc., whatever. There is a deal of humbug about this gynecological business!

The paper, printing, illustrations, and binding are all that can be desired.

So let the new edition of Brother Southwick's Gynecology take its course, do all the good it can, till an improved third edition is called for or till the book is replaced by a better one. W. S.

